# Changes in B Cell Pool of Patients With Multibacillary Leprosy: Diminished Memory B Cell and Enhanced Mature B in Peripheral Blood

**DOI:** 10.3389/fimmu.2021.727580

**Published:** 2021-09-21

**Authors:** Otto Castro Nogueira, Mariana Gandini, Natasha Cabral, Vilma de Figueiredo, Rodrigo Nunes Rodrigues-da-Silva, Josué da Costa Lima-Junior, Roberta Olmo Pinheiro, Geraldo Moura Batista Pereira, Maria Cristina Vidal Pessolani, Cristiana Santos de Macedo

**Affiliations:** ^1^Cellular Microbiology Laboratory, Oswaldo Cruz Institute, Oswaldo Cruz Foundation, Rio de Janeiro, Brazil; ^2^Immunoparasitology Laboratory, Oswaldo Cruz Institute, Oswaldo Cruz Foundation, Rio de Janeiro, Brazil; ^3^Leprosy Laboratory, Oswaldo Cruz Institute, Oswaldo Cruz Foundation, Rio de Janeiro, Brazil; ^4^Center for Technological Development in Health, Oswaldo Cruz Foundation, Rio de Janeiro, Brazil

**Keywords:** *Mycobacterium leprae*, B lymphocytes, B cell differentiation, active immune response, erythema nodosum leprosum

## Abstract

Despite being treatable, leprosy still represents a major public health problem, and many mechanisms that drive leprosy immunopathogenesis still need to be elucidated. B cells play important roles in immune defense, being classified in different subgroups that present distinct roles in the immune response. Here, the profile of B cell subpopulations in peripheral blood of patients with paucibacillary (TT/BT), multibacillary (LL/BL) and erythema nodosum leprosum was analyzed. B cell subpopulations (memory, transition, plasmablasts, and mature B cells) and levels of IgG were analyzed by flow cytometry and ELISA, respectively. It was observed that *Mycobacterium leprae* infection can alter the proportions of B cell subpopulations (increase of mature and decrease of memory B cells) in patients affected by leprosy. This modulation is associated with an increase in total IgG and the patient’s clinical condition. Circulating B cells may be acting in the modulation of the immune response in patients with various forms of leprosy, which may reflect the patient’s ability to respond to *M. leprae.*

## Introduction

Leprosy is a chronic infectious disease caused by the intracellular pathogen *Mycobacterium leprae*, which is endemic in many countries: in 2019, WHO reported 202,185 new cases worldwide (1, 2). The disease presents a complex clinical and immunopathological spectrum: at one end, tuberculoid leprosy (TT), in which skin lesions are characterized by a classical epithelioid cell granuloma formation, with a strong pro-inflammatory Th1/Th17 cellular immune response and consequent bacterial growth control. On the opposite side, lepromatous leprosy (LL), is characterized by a complete absence of granuloma and epithelioid cells in active lesions, the presence of humoral immune response and high bacillary load. Between these poles, there are unstable borderline forms: borderline tuberculoid (BT), borderline borderline (BB), and borderline lepromatous (BL) (3). For clinical diagnosis and treatment, WHO implemented an operational classification according to the number of skin lesions: paucibacillary (PB), patients with leprosy who present less than five lesions (tuberculoid); and multibacillary (MB), with five or more lesions (lepromatous) (4).

Patients with leprosy may present peripheral nerve demyelination and axonal loss, resulting in an impaired neural function, disfiguration, and deformities (5, 6). Leprosy reactions are acute inflammatory episodes that may occur at any stage of the clinical course of the disease (these reactions may affect 30–50% of all patients with leprosy). The most common episodes are Type I reaction (or reversal reaction-RR) and Type II reaction (also known as erythema nodosum leprosum-ENL). Expression levels of immunoglobulin receptors and B cell receptors during RR and ENL, evaluated by a transcriptomic analysis of peripheral blood mononuclear cells (PBMCs), support an antibody-mediated immune response during both RR and ENL (7). ENL is frequently associated with an intense infiltrate of neutrophils in the profound dermis and hypodermis, accompanied by macrophages, but skin fragments collected after 72 h of the reaction demonstrated the presence of lymphocytes, plasma cells, and mast cells (8).

The bacillus, and presumably similar breakdown products, are involved in the onset of the reactional episodes. Phospholipids are found in lepromatous tissues, as well as other bacillary breakdown products (9, 10) which could contribute to the stimuli of humoral responses in patients with LL. B-cells are activated by microorganisms *via* antigen-specific B-cell receptors (BCR) or non-specific pattern recognition receptors. The main mechanisms leading to antibody production by B cells are largely known and, Toll-like receptor (TLR) stimulation in B cells are associated with the regulation of the magnitude of the antibody response and the amount of antigen required for initiating BCR signaling (11, 12).

Antibody responses to specific *M. leprae* antigens have been used to diagnose patients affected by leprosy. The antibody titers generally increase as the disease progresses across the spectrum, from the TT to LL form. Patients affected by ENL also present higher titers of antibodies. In addition, the bacterial index is positively correlated with the antibody titers (13, 14).

The study of immune cells involved in leprosy immunopathogenesis is fundamental to understanding the phenomena that drive the evolution of subclinical to active leprosy (15), and several studies demonstrated that there is a significant increase in the risk of leprosy in contacts with an anti-PGL-I (anti-phenolic glycolipid-I) seropositivity (16, 17). PGL-I, despite its extreme lipophilicity due to its inherent phthiocerol dimycocerosyl component, is highly antigenic evoking high title IgM antibodies in patients affected by LL, attributable largely to the unique 3,6-di-O-methyl-beta-D-glucosyl entity at the non-reducing terminus of its trisaccharide (18). In the LL form of the disease higher titers of antibodies, complement and B-cell-derived IL-10 are observed, although it is not clear if it is responsible for the increased susceptibility in patients affected by LL (19–21). Additionally, IgG immune complexes are associated with the pathogenesis of ENL (22).

Although the relevance of innate and cellular immune responses in the pathogenesis of leprosy, several data suggest the involvement of B cells (humoral response) not only in reactional episodes, but in the pathogenesis of the disease. There are only a few publications about phenotypic analysis of peripheral B cells, restricted to some clinical presentations: Negera et al. studied the total count and frequencies of naïve, mature, and memory (resting, activated, and tissue-like) B cells in patients with ENL (23). Other authors compared the percentage of total B and of B1a cells, which are associated with autoimmune diseases, between patients with LL and uninfected subjects, and found that both are higher in the former (24). Tarique et al. found a higher frequency of B regulatory cells in antigen-stimulated PBMC of MB patients in comparison to PB and uninfected subjects (25). The pathways leading to B cell activation in leprosy are still unknown. Here, we analyzed and compared different B cell phenotypes in leprosy (multibacillary, paucibacillary and erythema nodosum leprosum) to elucidate a possible role of these B cells in the pathology of the disease.

## Materials and Methods

### Patients With Leprosy and Uninfected Subjects

Patients with leprosy were recruited from Souza Araújo Leprosy Outpatient Unit (Oswaldo Cruz Foundation, Rio de Janeiro-RJ, Brazil) from 2016 through 2019. Uninfected subjects, all residents in the city of Rio de Janeiro (State of Rio de Janeiro, Brazil), were selected according to the similarity of age (18 to 65) and gender patient’s cohort. The patients were classified on the leprosy spectrum clinically and histologically based on Ridley-Jopling classification schemes (26). The present study comprised 55 voluntary participants divided into four groups of donors: i) Patients with paucibacillary-PB (TT/BT) leprosy recruited before the start of multidrug therapy (MDT); ii) Patients with multibacillary-MB (LL/BL) recruited before the start of MDT with no signs of leprosy reactions at the time of leprosy diagnosis; iii) Patients clinically diagnosed with erythema nodosum leprosum-ENL (diagnosed - without treatment); iv) Uninfected subjects (**Table 1**; detailed information about patients and assays on **SI Table 1**). Patients and uninfected subjects with chronic or acute diseases unrelated to leprosy, diagnosed with other infectious diseases, using immunosuppressive drugs, or during pregnancy were excluded.

**Table 1:** Baseline characteristics of patients and uninfected individuals whose B cells were analyzed by flow cytometry.

Characteristics	Leprosy N = 35	PB N = 13	MB N = 12	ENL N = 10	Uninfected individuals N = 20
**Mean age (Years)**	47.9	48.7	47.5	45.8	36.53
**Gender, males (%)**	21 (60)	5 (38)	8 (66)	8 (80)	14 (70)
**Gender, females (%)**	14 (40)	8 (62)	4 (34)	2 (20)	6 (30)
**Mean Logarithmic Index of Bacilli (LIB)**	–	0	4,61	3,66	–
**Mean Bacilloscopic Index (BI)**	–	0	4,15	3,95	–

Groups included in this study: Leprosy: All patients, PB, Paucibacillary; MB, Multibacillary; ENL, erythema nodosum leprosum; and uninfected individuals.

### Ethics Statement

The use of samples was approved by the FIOCRUZ Research Ethics Committee (CAAE 01247418.8.0000.5248). All participants, including parents of minors, provided informed written consent.

### Isolation of the Peripheral Blood Mononuclear Cells and Flow Cytometry Staining

Blood samples were layered on Ficoll-Hypaque (Sigma Aldrich, USA) and mononuclear cells were isolated by centrifugation at 900 x *g* for 30 min, then stained with surface antibodies anti-human -CD3 (ALX 700, clone:UCHT1, Biolegend); -CD19 (APCCy7, clone SJ25C1, BD); -CD38 (PerCP-C5.5, clone HIT2, BD); -CD24 (FITC, clone ML5, BD); -CD27 (PECY7, clone 1A4CD27, Beckman Coulter); -CD21 (PECY7, clone B-ly4, BD); -IgM (BV510, clone g20-127, BD) and- IgD (PE, clone IA6-2, Beckman Coulter) for 30 min at 4°C in the dark. Cells were fixed with 2% paraformaldehyde and stored at 4°C. Data were collected using FACSAria IIup (BD Biosciences) and analyzed using FlowJo software (BD Biosciences) (17).

### ELISA

The IgG levels were determined in all plasma samples using an in-house ELISA. Briefly, MaxiSorp 96-well-plates (Nunc, Rochester, NY, USA) were coated with PBS containing 1.5 μg/ml of anti-human IgG (A0170, Sigma). After overnight incubation at 4°C, the plates were then washed three times with phosphate-buffered saline-0.05% Tween 20 (PBS-Tween) and blocked for 1 h at 37°C with PBS-Tween containing 5% nonfat-dried milk (PBS-Tween-M). Plasma samples diluted 1:1000 in PBS-Tween-M were added in duplicate wells containing 5% nonfat-dried milk (PBS-Tween-M) were added in duplicate wells. After 1 h at 37°C and three washings, specific antibodies were detected by goat peroxidase-conjugated anti-human IgG (Sigma, St. Louis) and followed by the addition of 3,3′,5,5′-tetramethylbenzidine (TMB) for 30 minutes. The reaction was stopped with HCl 1M (Merck) and optical density was measured at 450 nm using a SpectraMax microplate spectrophotometer (Molecular Devices, Sunnyvale, CA, USA). The IgG level in each sample was calculated interpolating the mean optical density value of the sample on a linear regression graphic of recombinant IgG (ref. 15260) standard curve dilution (ranging from 0.500 to 0.031 µg/mL) performed using the same conditions described above (27).

### Statistical Analysis

Differences in percentages of B cells, B cell subsets, and ELISA were analyzed using the Kruskal-Wallis test. Graphs were produced by GraphPad Prism version 8.0 for Mac (GraphPad Software, CA, USA). The statistical significance level was set at p<0.05; p<0.005; p<0.0005.

## Results

### Frequency of Different B Cell Subpopulations in PBMCs From Patients With Leprosy

**Figure 1:**
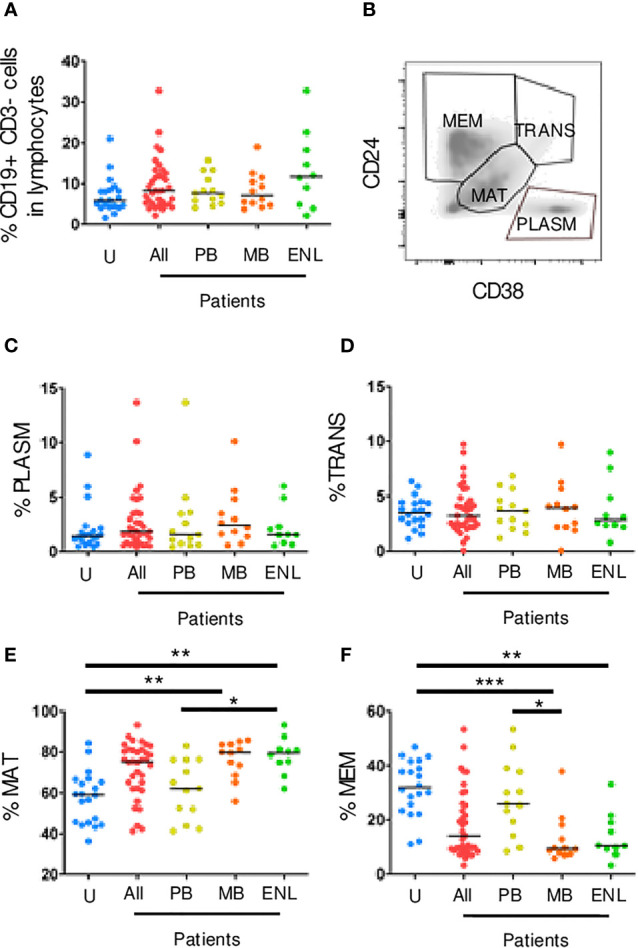
Circulating CD19+ B cell subpopulations sorted by CD24 and CD38 expression. PBMC of uninfected individuals (U), paucibacillary patients (PB), multibacillary patients in type II reaction (ENL) or not (MB) were freshly stained and analyzed by flow cytometry. **(A)** Frequency of CD19+ CD3- B cells among lymphocytes for all groups described above in which All represents PB, MB, and ENL. **(B)** Density plot profile for B cell subpopulations based on CD24 and CD38 expression in which memory B cells (MEM), transitional B cells (TRANS), mature B cells (MAT), and plasmablasts (PLASM) are depicted. Percentage of plasmablasts **(C)**, transitional B cells **(D)**, mature B cells **(E)**, and memory B cells **(F)** among CD19+ CD3- B cells. Each dot represents one donor and lines represent median values for each group. Significant values (*) were calculated by Kruskal-Wallis tests with Dunn’s multiple comparisons test. *p < 0.05; **p < 0.005; ***p < 0.0005.

To study peripheral B cell subpopulations, initially, lumps and monocytes were excluded using the parameters of frontal dispersion measured by area (FSC-A, forward scatter-area), versus frontal dispersion measured by height (FSC-H, forward scatter-height). Next, the lymphocyte region was selected by FSC-A and lateral dispersion measured by SSC-A area (side scatter), and in this region B cells (CD3-CD19+) were detected. The B cell subpopulation gate strategies were applied to different leprosy manifestations and uninfected subjects. No difference between the different clinical forms and uninfected subjects was observed (**Figure 1A**). Using this strategy, it was possible to identify four B cell subpopulations: memory B cells (CD24++CD38-/+), transitional B cells (CD24hiCD38hi), plasmablasts (CD24-CD38+) and mature B cells (CD24intCD38int) (**Figure 1B**).

The transitional B cells and plasmablasts can also produce IL-10 and regulate CD4+ T cell proliferation and differentiation toward T helper (Th) effector cells (28). Our results showed that there were no significant differences between patients and uninfected subjects in both transitional and plasmablast B cells (**Figures 1C, D**). Besides that, a larger frequency of mature B cells was observed in patients with MB leprosy (mean_79.70; range_30.10; p=0.0032) and ENL (mean_79.40; range_31.10; p=0.0030) in comparison to uninfected subjects. Patients with PB leprosy (mean_62.20; range_ 41.80; p=0.0450) in comparison to ENL patients (differences between MB e PB were not statistically significant) (**Figure 1E**). Memory B cells are formed within the germinal centers from mature cells. These cells also express higher affinity B cell receptors, which not only strengthens the effector functions of the antibodies secreted by their plasma cell progeny but also allows memory B cells to sense very low antigen levels. A decrease in the frequency of memory B cells in patients with MB leprosy, who present a higher bacillary load (mean_9.37; range_31.98; p=0.0002) and ENL (mean_10.50; range_29.90; p=0.0035) was observed in comparison to uninfected subjects. This decrease was also seen in patients with MB leprosy compared to those with PB leprosy (p=0.0163) (**Figure 1F**).

### Frequency of Memory B Cells in PBMCs From Patients With Leprosy

**Figure 2:**
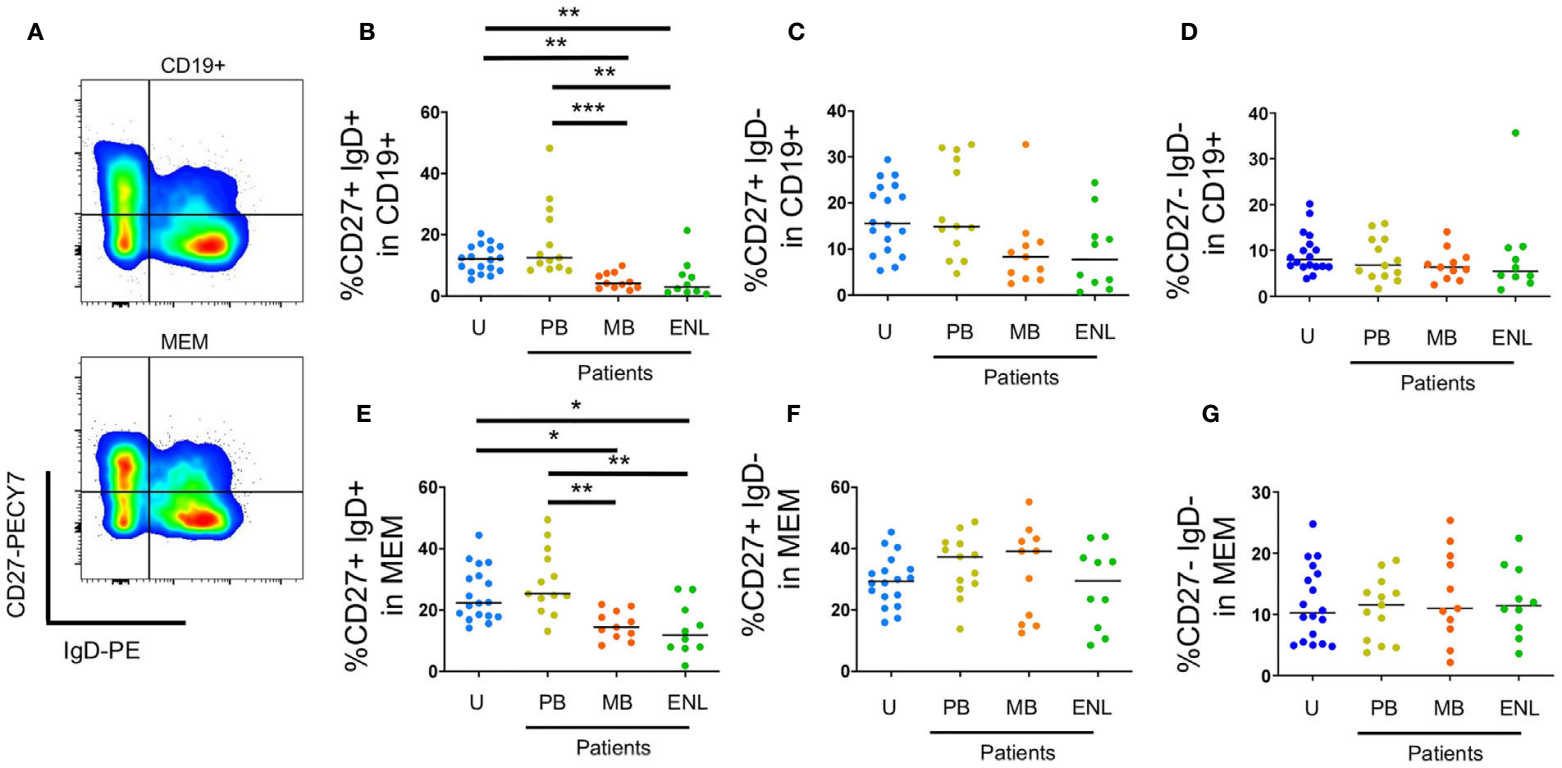
Circulating memory B cells sorted by CD27 and IgD. PBMC of uninfected individuals (U), paucibacillary patients (PB), multibacillary patients in type II reaction (ENL) or not (MB) were freshly stained and analyzed by flow cytometry. **(A)** Pseudocolor plot profile for CD19+ B cells (top) and MEM B cells (bottom) for CD27 and IgD expression. Percentage of CD27+IgD+ **(B)**, CD27+IgD- **(C)** and CD27-IgD- **(D)** cells among CD19+ B cell subpopulation or MEM B cell subpopulation (**E–G**, respectively). Each dot represents one donor and lines represent median values for each group. Significant values (*) were calculated by Kruskal-Wallis tests with Dunn’s multiple comparisons test. *p < 0.05; **p < 0.005; ***p < 0.0005.

Unswitched memory B cells (CD19+CD27+IgD+) are important in the first line of defense against infections because of the quick production of low-affinity IgM (29). **Figure 2A** is a gating strategy for selecting circulating memory B cells sorted by CD27 and IgD. Our results showed that there was a decrease in the frequency of unswitched memory B cells both in CD19+ B cells (CD27+IgD+) of patients with MB leprosy (p=0.0064)/ENL (p=0.0095) in comparison to uninfected subjects and patients with MB leprosy (p=0.0007)/ENL (p=0.0010) in comparison to patients with PB leprosy. This decrease was also seen within B memory cells (CD19+CD24+CD38+) in patients with MB leprosy (p=0.0308)/ENL (p=0.0222) in comparison to uninfected subjects and patients with MB leprosy (p=0.0036)/ENL (p=0.0026) in comparison to patients with PB leprosy (**Figures 2B, E**).

We did not observe differences in switched memory B cells between the different clinical profiles of the disease (**Figures 2C, D, F, G**). Resting memory B cells (CD19+CD21+CD27+) can produce antibodies in absence of T cell help, are highly proliferative, and have increased cell turnover compared to other B cell memory subpopulations (atypical memory and activated memory).

**Figure 3:**
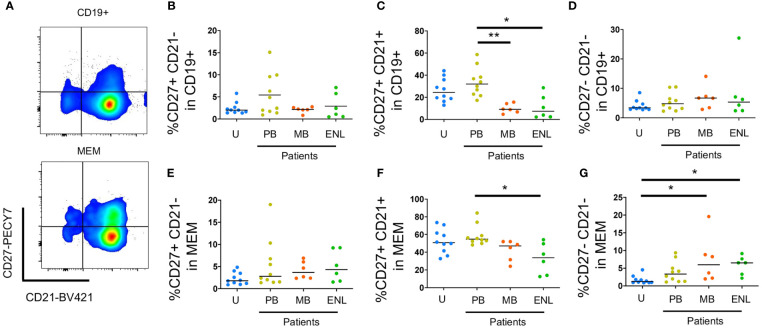
Circulating B cells sorted by CD21 and CD27. PBMC of uninfected individuals (U), paucibacillary patients (PB), multibacillary patients in type II reaction (ENL) or not (MB) were freshly stained and analyzed by flow cytometry. **(A)** Pseudocolor plot profile for CD19+ B cells (top) and MEM B cells (bottom) for CD21 and CD27 expression. Percentage of CD27+CD21- **(B)**, CD27+CD21+ **(C)**, and CD27-CD21- **(D)** cells among CD19+ B cell subpopulation or MEM B cell subpopulation (**E–G**, respectively). Each dot represents one donor and lines represent median values for each group. Significant values (*) were calculated by the Kruskal-Wallis test with Dunn’s multiple comparisons test. *P < 0.05; **p < 0.005; ***p < 0.0005.

**Figure 3A** shows the gating strategy for selecting circulating memory B cells sorted by CD27 and CD21 (30). Our data did not show significant differences between clinical forms and uninfected subjects in both B cells total and B memory cells (**Figures 3B, E**). We observed a reduced frequency in patients with MB leprosy and with ENL both in total B cells (CD19+) and in memory cells (CD19+CD24+CD38+/-) (**Figures 3C, F**) (23). No significant differences on atypical memory B cells (CD19+CD21-CD27-) in total B cells (CD3-CD19+) were observed (**Figure 3D**). However, we observed an increase in expression (CD19+CD21-CD27-) within memory B cells in patients with MB leprosy (p=0.0194) and with ENL (p= 0.0173) in comparison to uninfected subjects (**Figure 3G**).

**Figure 4:**
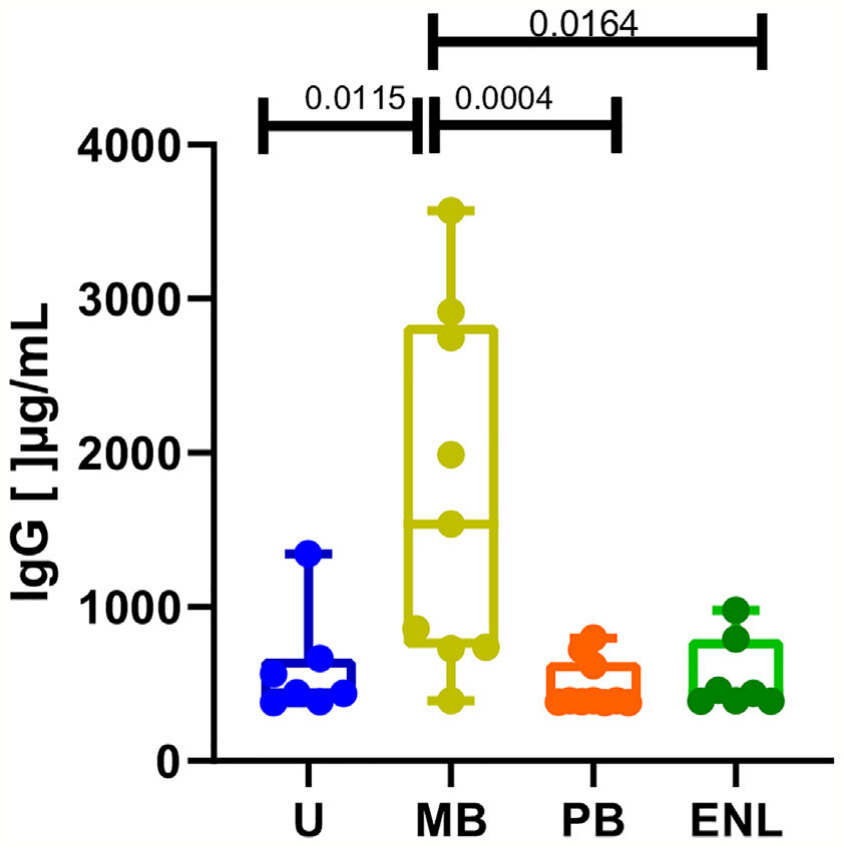
Total IgG levels measured by ELISA in sera from uninfected individuals (U), paucibacillary patients (PB), multibacillary patients in type II reaction (ENL) or not (MB). Significant values were calculated by the Kruskal-Wallis test with Dunn’s multiple comparisons test. A p-value < 0.05 was considered statistically significant.

Lastly, total IgG levels were assessed to corroborate the distinct profile of IgG antibody production in different clinical scenarios. The median of IgG levels in the uninfected subject group was 442.5 (range _ 966.1), 1539 in MB (range _ 3179), 319.7 in PB (IQ range _ 419.5), and 438.5 in ENL (range _ 589.4). Despite the relatively low number of analyzed donors, a significantly higher level of circulating IgG in patients with MB leprosy compared to PB (p=0.0004) and patients with ENL (p=0.0164) was observed (**Figure 4**). Moreover, all other groups included in our study (PB, ENL, and uninfected subjects) presented very similar levels of circulating IgG.

## Discussion

Leprosy is a complex and multifactorial disease in which the immune system is pivotal for determining the clinical course of the disease. Although several studies reinforce the importance of T cells in the pathogenesis of the disease, as well as genetic factors and the innate immune cells, the pathogenesis of leprosy is still not fully understood.

*M. leprae*-specific immunoglobulin G1 (IgG1) antibodies in patients with leprosy show a direct correlation with bacterial load suggesting that IgG1 B-cell responses may be surrogate markers of disease progression, although the role of B cells in the different clinical forms of the disease needs to be elucidated. There are still only few reports about the role of B cells in active leprosy lesions in different spectral forms of the disease, although there are several evidences of the involvement of B cells not only in the onset of reactional episodes but also in the course of non-reactional leprosy.

Fabel and colleagues (31) have suggested that B cells might be implicated in tuberculoid granuloma formation and type 1 reactions. They demonstrated that tuberculoid leprosy shows more B cells and less plasma cells than lepromatous leprosy. Here, we observed that there were no significant differences between patients and uninfected subjects in both transitional and plasmablast B cells, but a higher frequency of mature B cells was observed in both groups of patients with MB and ENL.

We acknowledge that one limitation of the present study is the absence of functional analysis but a previous morphometric analysis of B cells and plasma cells in ENL demonstrated a positive correlation with CD3-positive pan T cells in the biopsy and a negative correlation with T regulatory – T cell ratio (32).

Negera and colleagues reported that mature and memory B cells on patients with MB leprosy have no different frequencies from the ones in ENL (23). However, our data showed that, during ENL, atypical B cells (CD27-CD21-) decreased whereas activated B cells (CD27+CD21) increased in frequency. Despite the lack of consensus, those two subpopulations of memory B cells differ in function: activated B cells are readily prone to BCR reactivation and atypical B cells may represent an exhausted/anergic phenotype or a normal lineage of B cells with repeated antigen encounters (33).

In addition, CD27+CD21+ resting memory B cells were diminished in patients with MB leprosy, with only a slight significant increase in atypical memory B cells. Several reports point toward an increased subpopulation of atypical memory B cells in diseases with chronic antigen stimulation and many display a robust circulation of proinflammatory mediators (34, 35). However, multibacillary leprosy displays several anti-inflammatory mechanisms that favor pathogen persistence (15). It is well established that lipid mediators derived from the metabolism of ω3 and ω6 polyunsaturated fatty acids (PUFAs) are present in leprosy and its reactions and may play important roles in the modulation of the innate and adaptive immune responses (15, 36): patients with MB leprosy have higher levels of lipid mediators (prostaglandin E_2_, leukotriene B_4_, lipoxin A_4_) when compared to PB, and these substances can inhibit B cell activation (37–39). Furthermore, it is known that lipoxin A_4_ can inhibit the production of specific memory B cell antibodies (38). Because only a slight increase in the frequency of atypical memory B among the pool of total memory B cells was observed, we hypothesize that these anti-inflammatory mechanisms may impact memory B cell activation counterbalancing a high antigen availability with low or refractory T cell help. Also, the expansion of mature B cells may be the reason why atypical memory B cells were only statistically significant when analyzed in the memory cell pool. Our future studies will aim at the identification of which memory B cells are diminished or whether all phenotypes are equally diminished in patients with MB leprosy.

CD27+IgD+IgM+ memory B cells are also called marginal zone B cells and are pivotal to the response against encapsulated bacteria (40). Here, a great reduction in this subpopulation was observed in the multibacillary group. Indeed, some authors report that *M. leprae* (41) and cell wall components (42) are present in the blood. This high antigen availability may promote an enhanced plasma cell differentiation chronically in a manner that no great impact is immediately observed on plasmablast frequencies, for changes in this compartment are transient and detected easily during acute infections or vaccination (40).

Circulating memory B cells may also undergo apoptosis or be redistributed to other compartments. For instance, B cells are detected frequently in LL/BL lesions but sporadically observed in BT granulomas (43), in which both mature and plasma cells were found. Another hypothesis for the reduced frequencies of circulating memory B cells could be inferred from the high levels of total IgG observed in patients with MB leprosy. A state of polyclonal activation would activate memory B cell clones both nonspecific and *M. leprae*-specific to differentiate into plasma cells and increase the production of antibodies by the bone marrow resident cells. Indeed, the hypothesis of hypergammaglobulinemia in leprosy was already suggested by other groups (44, 45), although no specific antigen was implicated. Recently, our group showed that bacterial histone-like protein (hlp) and human DNA were increased in patients with MB leprosy when B cells also had increased levels of TLR9 expression (46). One of the mechanisms of maintenance of antibody levels is the induced recall by small quantities of bacterial DNA which promotes antibody production of all specificities and all subclasses (47). This serological memory is kept by bone marrow-resident plasma cells and by memory B cells, which are constantly restimulated by bystander cytokines and TLR-triggering on B cells in an antigen-independent way (48). Furthermore, both memory and naïve B cells could acquire plasma-cell phenotype *in vitro* after CpG stimulation (49). However, the persistence of *M. leprae*-derived circulating antigens, especially the bacterial DNA-histone complex, could impact memory and naïve B cell subpopulations. Therefore, we hypothesized that a state of polyclonal B cell activation by bacterial compounds would promote mature B cell expansion and migration of memory B cells to bone marrow or infection sites, reflected in increased numbers of the former and diminished numbers of the latter in the circulating pool.

B cell homeostasis is a highly regulated process, in which bone marrow is constantly producing B cells. Circulating B cells and soluble factors are responsible for B cell frequency maintenance in the peripheral tissues (50). Patients with MB leprosy have increased circulating mature B cells, however, no impact in transitional B cells was reported. Because the latter represents recent egress cells from bone marrow, higher production of B cells would impact transitional B cell frequency (51). Therefore, we cannot exclude the hypothesis that an increased production of survival factors in the periphery may diminish naïve B cell death.

Type II reaction or ENL is a pathological process that may be related to the break of *M. leprae* tolerance caused by MDT treatment and viral infections, for example (52). It results in a systemic inflammation to the bacillus which is often recurrent or chronic and is treated with corticosteroid or thalidomide (22). Negera et al. demonstrated that memory B cells are impacted after ENL treatment in paired samples, suggesting that a controlled inflammation has a role in B cell pool frequencies. As bacterial killing is enhanced in patients with ENL (53), there is a consequent increase in antigen availability, however, no differences in all B cell frequencies among patients with MB and ENL were observed here, but in total IgG levels. Those could be explained by the increased formation of immune complexes during ENL because of the high antigen availability (54).

The presence of different functionally active B-cell stages within lesions of patients with leprosy, including BT patients, which could secrete anti-*M*. leprae-specific antibodies were described (43). Our data suggest that antigen availability that occurs in patients with high bacillary load (MB and ENL) may be associated with alterations in the frequency and function of B-cell subpopulations (mature and memory B cells). It remains to be clarified whether the impact on the B cell pool is directly contributing to the clinical state of patients with MB leprosy or is simply a consequence of a failure in the interferon gamma (IFN-γ) efficient response in those patients, especially because patients with PB leprosy who can contain bacterial spread show similar B cell frequencies as uninfected subjects. To the best of our knowledge, it is the first study that demonstrates these different B cell phenotypes in polar forms of leprosy and in ENL, which can contribute to elucidate the role of B cell phenotypes in the disease.

## Data Availability Statement

The raw data supporting the conclusions of this article will be made available by the authors, without undue reservation.

## Ethics Statement

The studies involving human participants were reviewed and approved by FIOCRUZ Research Ethics Committee (CAAE 01247418.8.0000.5248). Written informed consent to participate in this study was provided by the participants’ legal guardian/next of kin.

## Author Contributions

ON and MG designed the analyses, collected, and analyzed data, and wrote the manuscript. VF and NC contributed with data collection. RR-d-S and JL contributed with analysis tools. RP, GP, and MP designed the analyses and supervised experimental work. CM supervised and wrote the manuscript. All authors contributed to the article and approved the submitted version.

## Funding

This work was supported by The New York Community Trust/Heiser Program for Research on Leprosy and The National Institute of Allergy and Infectious Diseases of the National Institutes of Health (NIAID/NIH) under the award number RO1AI129835.

## Conflict of Interest

The authors declare that the research was conducted in the absence of any commercial or financial relationships that could be construed as a potential conflict of interest.

## Publisher’s Note

All claims expressed in this article are solely those of the authors and do not necessarily represent those of their affiliated organizations, or those of the publisher, the editors and the reviewers. Any product that may be evaluated in this article, or claim that may be made by its manufacturer, is not guaranteed or endorsed by the publisher.
