# Supplementary material for: Changes in B Cell Pool of Patients With Multibacillary Leprosy: Diminished Memory B Cell and Enhanced Mature B in Peripheral Blood
Source: Front Immunol. 2021 Sep 21;12:727580. doi: 10.3389/fimmu.2021.727580 (PMC8490736; doi:10.3389/fimmu.2021.727580)
Supplement: Supplementary file 1 [file Table_1.docx]

**Supplementary Table 1. Detailed baseline characteristics of the patients with leprosy and uninfected individuals included in each experiment of the study.**

| **Patient Code** | **Age** | **Sex** | **Ridley-Jopling Classification** | **BI** | **LBI** | **Experiment** | **Figure** |
| --- | --- | --- | --- | --- | --- | --- | --- |
| SD02 | 34 | F | U | ******* | ******* | CF | 1(A-F); 2(A-G); |
| SD03 | 29 | F | U | ******* | ******* | CF | 1(A-F); 2(A-G); |
| SD04 | 35 | M | U | ******* | ******* | CF | 1(A-F); 2(A-G); |
| SD06 | 44 | M | U | ******* | ******* | CF | 1(A-F); 2(A-G); |
| SD07 | 52 | F | U | ******* | ******* | CF | 1(A-F); 2(A-G); |
| SD08 | 30 | F | U | ******* | ******* | CF | 1(A-F); 2(A-G); |
| SD09 | 35 | M | U | ******* | ******* | CF | 1(A-F); 2(A-G); |
| SD10 | 30 | F | U | ******* | ******* | CF | 1(A-F); 2(A-G); |
| SD11 | 56 | F | U | ******* | ******* | CF | 1(A-F); 2(A-G); 3(A-G); |
| SD12 | 39 | M | U | ******* | ******* | CF, ELISA | 1(A-F); 2(A-G); 3(A-G); 4A |
| SD13 | 53 | M | U | ******* | ******* | CF | 1(A-F); 2(A-G); 3(A-G); |
| SD14 | 33 | F | U | ******* | ******* | CF | 1(A-F); |
| SD15 | 35 | M | U | ******* | ******* | CF | 1(A-F); |
| SD16 | 44 | F | U | ******* | ******* | CF, ELISA | 1(A-F); 2(A-G); 3(A-G); 4A |
| SD17 | 27 | F | U | ******* | ******* | CF | 1(A-F); 2(A-G); 3(A-G); |
| SD18 | 36 | F | U | ******* | ******* | CF, ELISA | 1(A-F); 2(A-G); 3(A-G); 4A |
| SD19 | 49 | F | U | ******* | ******* | CF, ELISA | 1(A-F); 2(A-G); 3(A-G); 4A |
| SD20 | 34 | F | U | ******* | ******* | CF, ELISA | 1(A-F); 2(A-G); 3(A-G); 4A |
| SD21 | 27 | F | U | ******* | ******* | CF | 1(A-F); 2(A-G); 3(A-G); |
| SD22 | 24 | F | U | ******* | ******* | CF | 1(A-F); 2(A-G); 3(A-G); |
| SDNF | 32 | M | U | ******* | ******* | ELISA | 4A |
| SDN MCJA | 35 | F | U | ******* | ******* | ELISA | 4A |
| BT01 | 51 | F | BT | 0 | 0 | CF, ELISA | 1(A-F); 2(A-G); 4A |
| BT02 | 21 | M | BT | 0 | 0 | CF, | 1(A-F); 2(A-G); |
| BT05 | 36 | M | BT | 0 | 0 | CF, ELISA | 1(A-F); 2(A-G);4A |
| BT07 | 48 | M | BT | 0 | 0 | CF, ELISA | 1(A-F); 2(A-G); 3(A-G); 4A |
| BT08 | 34 | F | BT | 0 | 0 | CF | 1(A-F); 2(A-G); 3(A-G); |
| BT09 | 51 | M | BT | 0 | 0 | CF | 1(A-F); 2(A-G); 3(A-G); |
| BT10 | 66 | F | BT | 0 | 0 | CF, ELISA | 1(A-F); 2(A-G); 3(A-G); 4A |
| BT11 | 56 | F | BT | 0 | 0 | CF | 1(A-F); 2(A-G); 3(A-G); |
| BT13 | 19 | F | BT | 0 | 0 | CF, ELISA | 1(A-F); 2(A-G); 3(A-G); 4A |
| BT14 | 55 | F | BT | 0 | 0 | CF, ELISA | 1(A-F); 2(A-G); 3(A-G); 4A |
| BT15 | 65 | F | BT | 0 | 0 | CF | 1(A-F); 2(A-G); 3(A-G); |
| BT16 | 59 | F | BT | 0 | 0 | CF | 1(A-F); 2(A-G); 3(A-G); 4A |
| BT17 | 65 | M | BT | 0 | 0 | CF | 1(A-F); 2(A-G); 3(A-G); |
| AMS18 | 35 | M | BT | 0 | 0 | ELISA | 4A |
| MRA19 | 46 | M | BT | 0 | 0 | ELISA | 4A |
| LL01 | 59 | F | LL | 5 | UNKNOWN | CF | 1(A-F); |
| LL02 | 25 | F | LL | 5 | 3,85 | CF | 1(A-F); 2(A-G); |
| LL03 | 49 | F | LL | 5 | 2,85 | CF, ELISA | 1(A-F); 2(A-G); 4A |
| LL04 | 54 | M | LL | 4,75 | UNKNOWN | CF | 1(A-F); 2(A-G); |
| BL05 | 48 | M | BL | 3,75 | 4,8 | CF, ELISA | 1(A-F); 2(A-G); 4A |
| LL08 | 35 | M | LL | 4,5 | 5,9 | CF, ELISA | 1(A-F); 2(A-G); 3(A-G); 4A |
| BL09 | 64 | M | BL | 5 | 3,6 | CF, ELISA | 1(A-F); 2(A-G); 3(A-G); 4A |
| BL10 | 61 | M | BL | 4 | 3,5 | CF | 1(A-F); 2(A-G); 3(A-G); |
| LL11 | 49 | M | LL | 4 | 2,3 | CF, ELISA | 1(A-F); 2(A-G); 4A |
| LL12 | 40 | M | LL | 5,85 | UNKNOWN | CF, ELISA | 1(A-F); 2(A-G); 3(A-G);4A |
| LL13 | 35 | M | LL | UNKNOWN | UNKNOWN | CF, ELISA | 1(A-F); 2(A-G); 3(A-G);4A |
| BL14 | 51 | M | BL | 3,25 | 4,6 | CF | 1(A-F); 2(A-G); 3(A-G); |
| MMS15 | 43 | F | BL | UNKNOWN | UNKNOWN | ELISA | 4A |
| ENL01 | 46 | M | ENL | 5 | 3,6 | CF, ELISA | 1(A-F); 2(A-G); 4A |
| ENL02 | 35 | M | ENL | 5,25 | 2,85 | CF | 1(A-F); 2(A-G); |
| ENL03 | 26 | F | ENL | 5 | 3,85 | CF, ELISA | 1(A-F); 2(A-G); 4A |
| ENL04 | 31 | M | ENL | 2,75 | 2,5 | CF | 1(A-F); 2(A-G); |
| ENL05 | 53 | M | ENL | 5 | 5,2 | CF | 1(A-F); 2(A-G); 3(A-G); |
| ENL06 | 56 | F | ENL | 4,5 | 4,8 | CF | 1(A-F); 2(A-G); 3(A-G); |
| ENL07 | 50 | M | ENL | 4.75 | 3.6 | CF, ELISA | 1(A-F); 2(A-G); 3(A-G); 4A |
| ENL08 | 75 | M | ENL | 2,75 | 4,5 | CF, ELISA | 1(A-F); 2(A-G); 3(A-G); 4A |
| ENL09 | 48 | M | ENL | 2,7 | 4,5 | CF, ELISA | 1(A-F); 2(A-G); 3(A-G); 4A |
| ENL10 | 38 | M | ENL | 0 | UNKNOWN | CF | 1(A-F); 2(A-G); 3(A-G); |
| ACS 11 | 42 | M | ENL | UNKNOWN | UNKNOWN | ELISA | 4A |
| APS 12 | 42 | M | ENL | UNKNOWN | UNKNOWN | ELISA | 4A |
|  |  |  |  |  |  |  |  |
| U, uninfected individuals; LL, lepromatous leprosy; BL, borderline lepromatous; BT, borderline tuberculoid; ENL, erythema nodosum leprosum; BI, bacillary index; CF, flow cytometry; LBI, logarithmic bacillary index of skin lesion; ELISA, enzyme-linked immunosorbent assay; F, female; M, male. | | | | | | | |
|  |  |  |  |  |  |  |  |
